# Seed Traits and Germination of Invasive Plant *Solanum rostratum* (Solanaceae) in the Arid Zone of Northern China Indicate Invasion Patterns

**DOI:** 10.3390/plants13233287

**Published:** 2024-11-22

**Authors:** Hailun Yu, Runxia Zhang, Wenda Huang, Wei Liu, Jin Zhan, Ruixiong Wang, Xueyong Zhao, Qi Feng

**Affiliations:** 1 Key Laboratory of Ecological Safety and Sustainable Development in Arid Lands, Northwest Institute of Eco-Environment and Resources, Chinese Academy of Sciences, Lanzhou 730000, China; yuhailun@nieer.ac.cn (H.Y.); liuw@lab.ac.cn (W.L.); 2 State Key Laboratory of Biocontrol, School of Ecology, Sun Yat-sen University, Guangzhou 510275, China; zhangrx63@mail.sysu.edu.cn; 3 Naiman Desertification Research Station, Northwest Institute of Eco-Environment and Resources, Chinese Academy of Sciences, Lanzhou 730000, China; huangwd@lzb.ac.cn (W.H.); zhanjin@nieer.ac.cn (J.Z.); wangrx@nieer.ac.cn (R.W.)

**Keywords:** response pattern, seed germination, alien species, invasion strategy

## Abstract

The ability of seeds to germinate under a wide range of environmental conditions is an important characteristic of invasive alien plant species. *Solanum rostratum* Dunal, has been widely distributed in the Northeast and Northwest of China and is causing huge damage to the local agricultural production. Studies on seed germination and response among populations to environmental stress may assist in revealing the adaptability of invasive plants and how they cope with climate change. In this study, we collected seeds from five invasive plant populations of *S. rostratum*, with intervals of over 3000 km between them, distributed in different habitats and climate zones. We measured the differences in seed traits between populations and studied the trends in germination responses of *S. rostratum* seeds under diverse abiotic stress conditions. The weight and size of *S. rostratum* seeds distributed in Northeast China were significantly greater than those distributed in Northwest China; for the response of *S. rostratum* seed germination to environmental factors, seeds from arid and extremely arid areas of Northwest China had greater tolerance to high temperatures and osmotic stress, while seeds from semi-arid areas of Northeast China were more sensitive to low temperatures and high salt stress. Overall, the germination of *S. rostratum* seeds responded differently to various environmental stress factors, reflecting the ability of *S. rostratum* to occupy germination sites under low resource competition. Given the rapid changes in the global climate, our findings provide new insights into the seed adaptation strategies of alien plants during the invasion process and the mechanisms involved.

## 1. Introduction

The invasion of alien plants represents an important threat to biodiversity conservation and ecosystem maintenance under global climate change [1,2,3]. Plant invasion is the result of complex interactions between biological factors (e.g., artificial pressures on alien reproductive systems and functional characteristics of alien plants) [4,5,6] and abiotic factors (e.g., resource availability and variability of the environment to be invaded) [7,8,9,10]. Rapid economic development and changes in climatic conditions are the primary drivers of plant invasion [11,12,13]. Numerous studies have shown that nonbiological factors such as climate can shape the diversity and variation in plant functional traits at a large spatial scale. Local adaptation of invasive species is an important factor in their adaptation to new environments, successful settlement, and dissemination [14,15,16,17]. Many invasive plants reproduce through seeds; therefore, seed traits are crucial for the survival of invasive plants. Germination is one of the key seed traits that determines the establishment and expansion of plant populations [18,19,20] as well as the timing and intensity of resource utilization and competition. Germination is a crucial link for the successful colonization and rapid spread of alien species, especially for widely distributed invasive species [21]. Successful germination may increase competitive advantages in establishing and recruiting populations under novel environmental conditions [22].

The importance of seed germination in the invasion potential of plants has been demonstrated in relevant research studies, and it is generally believed that characteristics, such as germination rate, germination time, and germination speed, play important roles in the invasion of alien plants [23,24]. The changes in seed traits between and within populations are key factors leading to the establishment and persistence of invasive plant species [25,26,27]. Seed traits are influenced by many factors, including genetic variation and the environmental conditions during seed development [26,28]. These factors collectively determine the persistence (dormancy), germination, diffusion, or death of the seeds produced by plants [28,29]. Seeds from different individuals within the same species group often exhibit variations in germination characteristics [30]. Due to inherent environmental conditions, seeds collected from different locations, altitude gradients, and habitats may also exhibit differences in seed germination biology [28,31,32]. The geographical variation in seed germination is significantly correlated with climate variables [33,34]. However, due to limited resources, there may be trade-offs between the seed characteristics of invasive plants. Therefore, as a response to different environmental conditions, invasive plant seeds may adopt different adaptation strategies during germination, leading to differences in seed germination characteristics [34,35]. Studying the adaptive changes in alien plant seed germination to heterogeneous habitats can help to reveal how invasive plants respond to changing environments [36]. This knowledge can ultimately deepen our understanding of species invasion mechanisms, more fully elucidate the invasive characteristics of alien species [37], and improve our ability to predict future plant distributions and range expansion.

The Solanaceae family is the largest of the Solanaceae, with many species widely distributed throughout the world. Since many cultivars of Solanaceae are an important part of the world’s food, they are prone to escape and invade natural habitats during introductory cultivation [38]. For example, *Solanum torvum* Sw. (Solanaceae), which is native to the West Indies and has been naturalized in several tropical and subtropical regions around the globe, has been found in wild populations in southern Italy [38]. Statistical research has shown that of the more than 515 species of invasive plants that have successfully invaded China, 24 species of invasive plants belonging to the Solanaceae family have been identified, and 7 species have been recognized as “serious invasive plants”, which means that nearly 30% of the invasive plants in the Solanaceae family have been distributed in at least one or more natural geographic areas in China; meanwhile, they have caused great losses or obvious impacts on the economy and in ecological settings [39]. Among these, *Solanum rostratum* Dunal (Solanaceae), which invaded Northern China and is now widely distributed, was included in the first batch of China’s National Key Management Exotic Invasive Species List in 2013. This plant has strong reproductive and competitive abilities, and it can thrive even in extremely arid desert habitats [40]. At the same time, *S. rostratum* can rapidly form dominant populations, destroying invaded ecosystems [41] and disrupting the species balance within communities [42]. *Solanum rostratum* is one of the key invasive plants that need to be monitored and controlled in arid and semi-arid areas of China. At present, *S. rostratum* is widely distributed in the northwest and northeast regions of China; it has been observed in different climatic regions and has shown a particularly strong invasive trend in Northeast China [43]. The main distribution areas of *S. rostratum* in China are more than 3000 km apart, with significant differences in habitat conditions. There have been many studies on the physiological and ecological characteristics of *S. rostratum* [40,44,45,46]. However, there is still a lack of information concerning the potential adaptive strategies for seed germination within the various populations of this plant.

To understand the adaptive strategies of different populations of *S. rostratum* seeds to different temperature, water, and salt stress environments during germination, we collected *S. rostratum* seeds from five populations in China and measured the responses of the seeds to temperature factors (including constant and variable temperatures), water stress, and salt stress during germination. We investigated the adaptive changes in seed traits and germination strategies as adaptations to different environmental stress factors at the community level and addressed the following questions: (i) Are there significant differences in seed traits among populations of *S. rostratum* in different areas? (ii) What are the differences in the responses to environmental stress factors during the germination of *S. rostratum* seeds in populations with different distributions? (iii) With the intensification of global climate change, how will the germination strategy of *S. rostratum* seeds respond?

## 2. Materials and Methods

### 2.1. Study Species

*Solanum rostratum* was initially discovered in Liaoning Province in 1981, and it has spread across a large area in Northern China over the last 39 years [41]. The species grows in provinces of China, including Liaoning, Jilin, Beijing, Hebei, Shanxi, Xinjiang Uyghur Autonomous Region (hereafter Xinjiang), and Inner Mongolia Autonomous Region (hereafter Inner Mongolia) [47]. The species occurs in waste places, along roadsides, on banks of rivers and canals, in land used for grazing, and in cultivated fields [48].

### 2.2. Sample Collection

**Figure 1:**
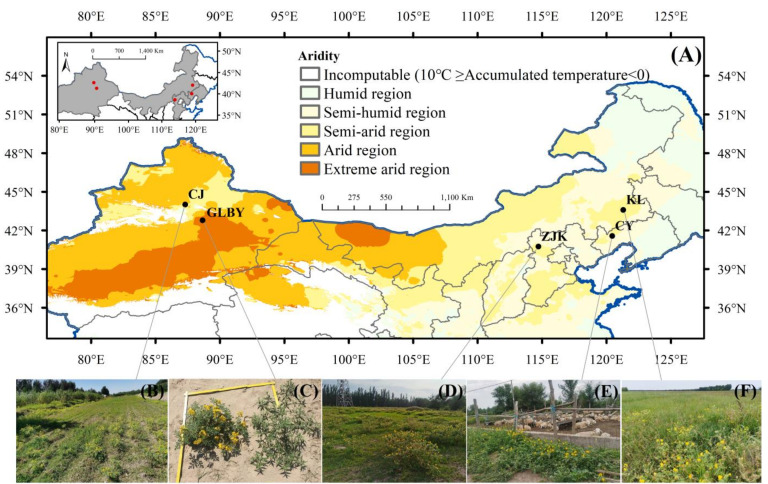
Sampling sites from five populations of *Solanum rostratum* Dunal (Solanaceae) in this research. (**A**) Geographic locations and climate of five samples; (**B**) collection site in Changji prefecture (abbreviated as CJ) in Xinjiang where distribution habitat is farmland; (**C**) collection site in GuoLebuyi township (abbreviated as GLBY) in Xinjiang where distribution habitat is barren land; (**D**) collection site in Zhangjiakou city (abbreviated as ZJK) where distribution habitat is arboretum; (**E**) collection site in Chaoyang city (abbreviated as CY) where distribution habitat is livestock pens; (**F**) collection site in Kailu County (abbreviated as KL) where distribution habitat is grassland communities.

From August 2019 to October 2019, sampling was performed in multiple regions of Hebei, Inner Mongolia, Liaoning, and Xinjiang according to different administrative divisions, natural conditions, and climate types. Plant communities with high habitat heterogeneity were selected, and five survey plots were set up (each plot measured 5 m × 5 m, with an interval of more than 5 km between plots) to ensure that the sampling areas covered the various distribution habitats, vegetation types, climate conditions, and soil conditions of *S. rostratum* as much as possible (Figure 1). Appendix A Table A1 lists specific geographic locations and climate information for the seed collection points. Climate zoning was based on the Chinese meteorological background dataset [49]. The temperature and rainfall data for each sampling site in 2018 were taken from the National Meteorological Science Data Center of China [50].

For all the collected samples, the seeds were cleaned and air-dried at room temperature and stored in a refrigerator at 4 °C for 15 weeks. Before the experiment began, the surfaces of *S. rostratum* seeds were disinfected with a 0.52% sodium hypochlorite solution for one minute, then rinsed 5–8 times with distilled water to avoid fungal infection. Thirty seeds were placed in a culture dish with a diameter of 9 cm containing two layers of sterilized filter paper, and 8 mL of distilled water was added. There were five replicate cultures for each experimental treatment. The experiment adopted a completely randomized design, with the positions of the culture dishes changing every day. The culture dishes were kept moist, and the results were recorded twice a day. Germination was considered when the seed embryonic root extended 2–3 mm, and the experiment ended when there was no seed germination for seven consecutive days. The experiment was conducted for a total of 30 days.

### 2.3. Measurement of Seed Size

We recorded the color, characteristics, and size of *S. rostratum* seeds. Seeds collected from each sample plot were evenly mixed, and 100 seeds were randomly selected as an analysis sample. An electronic balance (1/10,000, Statorius BS210S, Shanghai, China) was used to weigh the seeds, repeating the measurements 10 times each, to determine the mass of 1000 seeds. Fifty seeds were randomly selected from each sample, and seed images were captured using a high-speed scanner. The size of the seeds was calculated using Image J software. We calculated the average length and width of the seeds in each plot.

### 2.4. Effect of Temperature

To determine the effect of temperature on the germination of *S. rostratum* seeds from different distribution areas, we set up a constant temperature gradient (10, 15, 20, 25, 30, and 35 °C) experiment in a light incubator and a variable temperature gradient (15/5, 25/15, 30/20, and 38/28 °C) in another light incubator. Both temperature experiments were conducted under a 12 h/12 h alternating light and dark (environment). The thermostatic gradient simulated the monthly average temperature gradient experienced by different distribution areas of *S. rostratum* during a complete growing season. The variable temperature gradient simulated the monthly average temperature and extreme high-temperature values experienced by *S. rostratum* in different distribution areas during the peak growth and development period from April to July. Due to the widespread high temperature during the growing season of *S. rostratum* in Tuokexun County, higher temperature conditions were included in the experiment.

### 2.5. Effect of Salt Stress

To study the effect of salt stress on the germination of seeds of different populations of *S. rostratum*, NaCl solution was prepared and administered under optimal germination conditions (12 h light/12 h dark; 30/20 °C temperature). Five NaCl concentration gradients (0, 100, 200, 300, and 400 mM) were simulated and designated as no stress (a NaCl concentration of 0–100 mM), mild salt stress (100 mM), moderate salt stress (200 mM), severe salt stress (300 mM), and high-concentration salt stress (400 mM).

### 2.6. Effect of Osmotic Potential (Water Stress)

Because polyethylene glycol (PEG) is an inert substance that cannot penetrate the plant cell wall, we studied the effect of osmotic stress on the germination of *S. rostratum* seeds in different distribution areas by treating the seeds with PEG 6000 solution. The PEG concentration osmotic potential was calculated under established temperature conditions (12 h light/12 h dark and 20 °C constant temperature). PEG6000 solutions with osmotic potential of 0 (deionized water control), −0.21, −0.44, −0.75, and −1.0 MPa were prepared to test the effects of different water stress gradients on the germination of *S. rostratum* seeds.

### 2.7. Statistical Analysis

Appropriate measures must be employed to study the changes in germination strategy [51]. Several mathematical functions were used to calculate cumulative germination rates given count data and to describe the change trend in final germination rate with increasing stress levels through nonlinear regression analysis [52,53,54]. We conducted principal component analysis (PCA) on the mass, length, and width variables of *S. rostratum* seeds in different distribution areas using a “FactoMineR” software package. One-way analysis of variance (ANOVA) was performed for seed size and mass, with Tukey’s multiple comparison test used to compare the mean values between locations. The “germinationmetrics” package was used to calculate the final germination percentage (FGP) and mean germination time (MGT) of seeds from different geographical populations and for each treatment under temperature, osmotic, and salinity stress gradients. Two-way ANOVA was used to analyze differences in the final germination results. Before conducting the analysis of variance, the Shapiro–Wilk test was used to test for normality and Levene’s test was used to test the homogeneity of variance. The “lm” function from R version 4.0.0 [55] was employed to fit the mean germination time obtained in each stress experiment. Before analysis, the FGP was subjected to an inverse sine transform to ensure that the frequency distributions followed the normality assumption.

## 3. Results

### 3.1. Variation in Seed Traits Indicators and Correlations Between Different Distribution Areas

**Figure 2:**
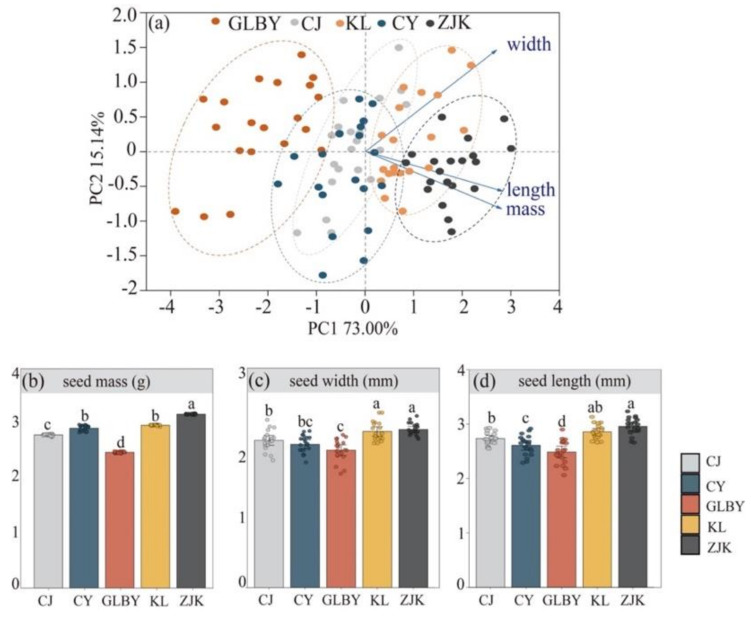
Principal component analysis (**a**) of seed weight, width, and length variables in different geographical populations of *Solanum rostratum* Dunal (Solanaceae) and the results of one-way analysis of variance (ANOVA) of the differences in seed traits among different distribution areas of *S. rostratum*. The first two PCA axes (**a**) represent the composite measures of “seed weight and length” and “seed width”. The results of one-way ANOVA characterize the differences in seed mass (**b**), seed width (**c**), and seed length (**d**) of *S. rostratum* in different areas. Different letters represent significant differences in seed traits between each distribution area. The error bars at the top of the histograms represent the standard error. Dots indicate the original sample data.

The surfaces of mature *S. rostratum* seeds are rough and uneven, densely covered with honeycomb-shaped concave network patterns, and the seed coat was thick and hard [56]. The color of the seeds of the five distribution areas of the *S. rostratum* in this study was black or dark brown, with no remarkable differences. In the principal component analysis (PCA), the first two axes explained the 73.00% and 15.14% variations in the total variation, respectively (Figure 2a). Seed mass, width, and length in the GLBY distribution area were significantly different between KL and ZJK; meanwhile, seeds from CJ were significantly different from ZJK, indicating that *S. rostratum* seeds from the Xinjiang populations (GLBY and CJ) differed from the seeds distributed in Northern and Northeast China (ZJK and KL). The mass and size of CY seeds were not significantly different from the other four distribution areas. The PCA indicated that the seed traits of *S. rostratum* in the different regions underwent adaptive changes to habitat conditions.

The results of the one-way ANOVA show that there were differences in seed mass, length, and width of *S. rostratum* from different distribution areas. The seed mass of *S. rostratum* from the ZJK area was the highest, significantly higher than seeds from other areas. The difference in seed mass between KL and CY was not significant; both were lower than in seeds from ZJK, but higher than in seeds from the CJ and GLBY areas. Seed mass in the GLBY area was significantly lower than in other areas (Figure 2b). The length and width of seeds collected from ZJK were greater than those from other distribution areas. The length and width of seeds from the KL area were lower than those from ZJK, but higher than those from other areas. There was no significant difference in seed size between CY and CJ. GLBY had the lowest values of seed length and width (Figure 2c,d) when compared to other areas.

### 3.2. The Seed Germination of S. rostratum from Different Areas in Response to Temperature Changes

#### 3.2.1. The Effect of a Constant Temperature Gradient on Seed Germination

**Figure 3:**
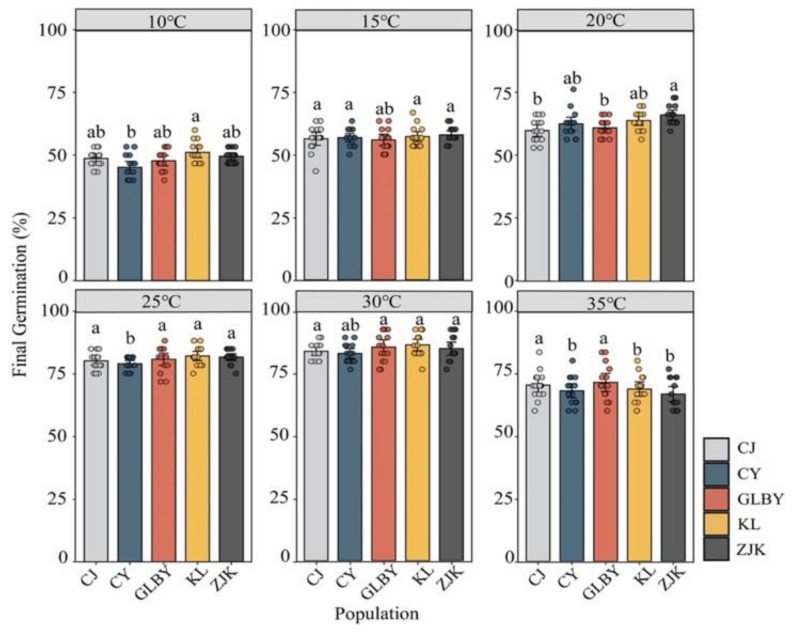
The effect of constant temperature gradients on the final germination rate of *Solanum rostratum* Dunal (Solanaceae) seeds from different distribution areas. Different letters represent significant differences in constant temperature conditions between each distribution area. The error bars at the top of the histograms represent the standard error. Dots indicate the original sample data.

Under constant temperature conditions, there were significant differences in the FGP of *S. rostratum* from different areas (*p* < 0.001; Figure 3, Table 1). Under a constant temperature of 30 °C, the *S. rostratum* FGP in each distribution area reached its maximum value, with the FGP exceeding 86%. The FGP in the KL distribution area exceeded 90%, and there was no significant difference in the FGP between distribution areas (*p* < 0.001). In low-temperature environments (10 °C), only the seeds from KL had an FGP exceeding 50% (51.11%) (*p* < 0.01). At 15 °C, the FGP of *S. rostratum* from all of the areas exceeded 55%, with the highest FGP in seeds from ZJK (57.78%) and the lowest in seeds from GLBY (55.78%) (*p* < 0.05). Under an ambient temperature of 20 °C, the FGP of *S. rostratum* from the three populations in the east (ZJK, KL, and CY) was higher than in seeds from two populations in the west (CJ and GLBY) (*p* < 0.01). At 25 °C, the FGP of *S. rostratum* from each area was significantly increased, exceeding 80%. The FGP in seeds from CY was the lowest (80.67%), significantly lower than in seeds from other areas (*p* < 0.001). At 35 °C, the FGP in seeds from the two western distribution areas (CJ, 70.22% and GLBY, 71.33%) was significantly higher than in seeds from the three eastern areas (KL, 68.67%; CY, 68%; and ZJK, 66.67%) (*p* < 0.001).

**Figure 4:**
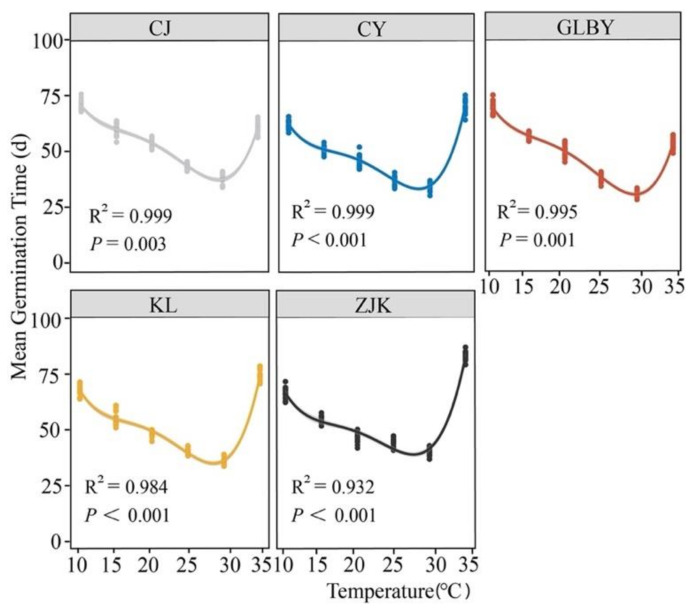
The effect of a constant temperature gradient on the average germination time of *Solanum rostratum* Dunal (Solanaceae) seeds from different areas. The lines represent the average time required to germinate within 30 days after the start of the experiment. The points represent the standard errors of the means.

The results for the mean germination time (MGT) of *S. rostratum* seeds from different areas to constant temperature changes (Figure 4) show that the MGT of *S. rostratum* seeds was significantly decreased with the increase in temperature, reaching a minimum at 30 °C. Under low-temperature conditions (10 °C), the MGT in the CJ and GLBY distribution areas was greater than seven days, while the MGT in the CY, KL, and ZJK distribution areas was less than seven days; under high-temperature conditions (35 °C), the mean MGT in seeds from CJ was close to six days, while the MGT in seeds from GLBY was 5.81 days. The MGT in the CY, KL, and ZJK distribution areas was greater than seven days, while the mean MGT in seeds from ZJK was 7.95 days. Specifically, when the temperature increased from 10 °C to 30 °C, the MGT of seeds from each area showed significant decreases. Under a temperature of 35 °C, the MGT values of CY (R^2^ = 0.999, *p* < 0.001), KL (R^2^ = 0.984, *p* < 0.001), and ZJK (R^2^ = 0.932, *p* < 0.001) were significantly increased. The slope of the curve of the MGT response to temperature changes in these areas was greater than that for seeds from the CJ distribution area (R^2^ = 0.999, *p* = 0.003) and GLBY distribution area (R^2^ = 0.995, *p* = 0.001).

#### 3.2.2. The Effect of Temperature Variation on Seed Germination

**Figure 5:**
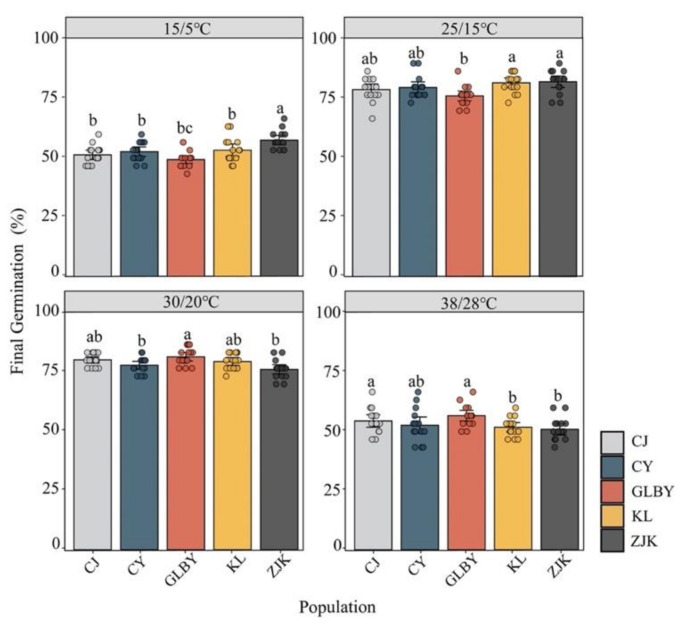
The effects of various constant temperature gradients on the final germination rate of *Solanum rostratum* Dunal (Solanaceae) seeds from different areas. Different letters represent significant differences in temperature variation conditions between each distribution area. The error bars at the top of the histograms represent the standard error. Dots indicate the original sample data.

The results of the two-way ANOVA indicate significant differences in seed germination rates among the areas (*p* < 0.001) (Figure 5, Table 1). Under variable temperature conditions of 15/5 °C, the FGP of *S. rostratum* seeds from ZJK reached 57.56%, significantly higher than the values of seeds from other areas. The FGP of *S. rostratum* seeds from GLBY did not exceed 50% (49.33%). Under temperature conditions of 25/15 °C, the FGP of seeds from KL (81.78%) and ZJK (82.22%) exceeded 80%, significantly higher than in seeds from GLBY (76.22%). At a temperature of 30/20 °C, the FGP values of seeds from GLBY (81.56%) and CJ (80.22%) exceeded 80%, significantly higher than in seeds from other distribution areas. Under these temperature conditions, the FGP of seeds from ZJK was only 76.22%. To study the differences in seed germination rates of *S. rostratum* from different areas under high temperatures, a 38/28 °C gradient was set up to simulate day and night high temperatures. The results show that the FGP of *S. rostratum* seeds from each area exceeded 50%, while GLBY (56.67%) and CJ (54.44%) seeds had a significantly higher FGP than seeds from KL (51.78%) and ZJK (50.89%). The germination rates of seeds from two sampling points in Xinjiang showed high temperature tolerance.

**Figure 6:**
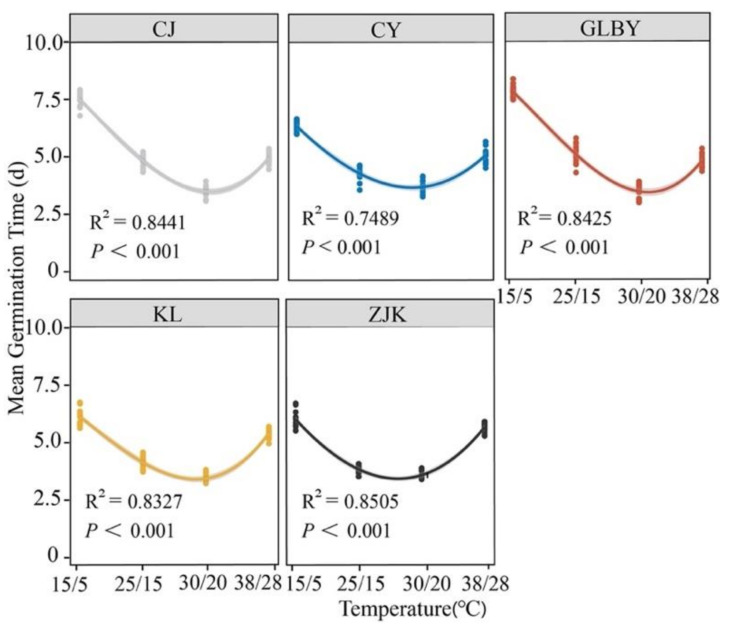
The effect of a constant temperature gradient on the average germination time of *Solanum rostratum* Dunal (Solanaceae) seeds from different areas. The lines represent the average time required for germination within 30 days after the start of the experiment. The points represent the standard errors of the means.

The responses of the MGT of *S. rostratum* seeds from different areas to a diurnal temperature gradient (Figure 6) show that under changing conditions, the MGT of *S. rostratum* seeds was significantly decreased with the increase in diurnal temperature. At 30/20 °C, the MGT of seeds from each area reached the minimum. Except for CY where the MGT of seeds was greater than 4 d (4.17 d), the MGT of seeds from other areas was less than 4 d. Under low day and night temperature conditions (15/5 °C), the MGT in seeds from CJ and GLBY was greater than 7 d, while the MGT in seeds from CY, KL, and ZJK was less than 7 d. Under high-temperature conditions (38/28 °C), the MGT of GLBY was less than five days, and the germination time was shorter. The MGT in seeds from CY, KL, and ZJK was greater than 5 d, while the MGT in seeds from ZJK was nearly six days (5.91 d). Overall, the slopes of the temperature response curves for the MGT in the seeds from the GLBY distribution area (R^2^ = 0.8425, *p* < 0.001) and CJ distribution area (R^2^ = 0.8441, *p* < 0.001) were greater than that in the CY (R^2^ = 0.7489, *p* < 0.001), KL (R^2^ = 0.8327, *p* < 0.001), and ZJK (R^2^ = 0.8505, *p* < 0.001) distribution areas. Under extreme high-temperature conditions, the MGT of seeds from the GLBY and CJ areas was lower than those from the other three areas, demonstrating strong resistance to extreme high temperatures.

### 3.3. The Effect of NaCl Concentration on the Germination of S. rostratum Seeds

**Figure 7:**
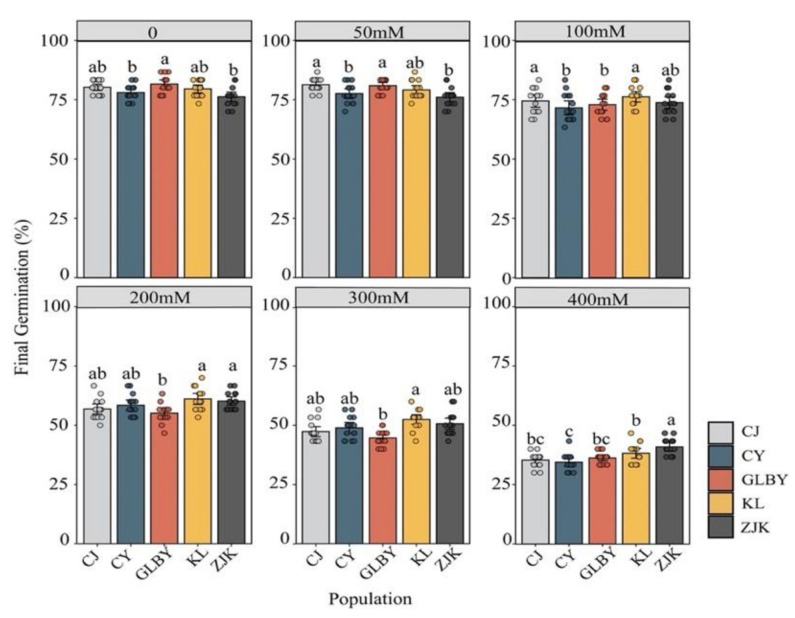
The effect of NaCl stress on the final germination rate of *Solanum rostratum* Dunal (Solanaceae) seeds from different areas. Different letters represent significant differences between each distribution area under the same NaCl stress conditions. The error bars at the top of the histograms represent the standard error. Dots indicate the original sample data.

The FGP of *S. rostratum* seeds from different areas significantly decreased with the increase in NaCl concentration (*p* < 0.001) (Figure 7, Table 1). The results of the two-way ANOVA show that under no stress (CK) and mild NaCl stress (50 mM), the FGP of *S. rostratum* seeds from GLBY and CJ exceeded 80%, significantly higher than in seeds from other areas. With the increase in the NaCl concentration (reaching 100 mM and 200 mM), the FGP of seeds from GLBY showed a downward trend, significantly lower than that of seeds from other areas under the same concentration. Under higher concentrations of NaCl (up to 400 mM), more than 40% of the seeds from ZJK successfully germinated, significantly higher than the FGP in seeds from other areas.

**Figure 8:**
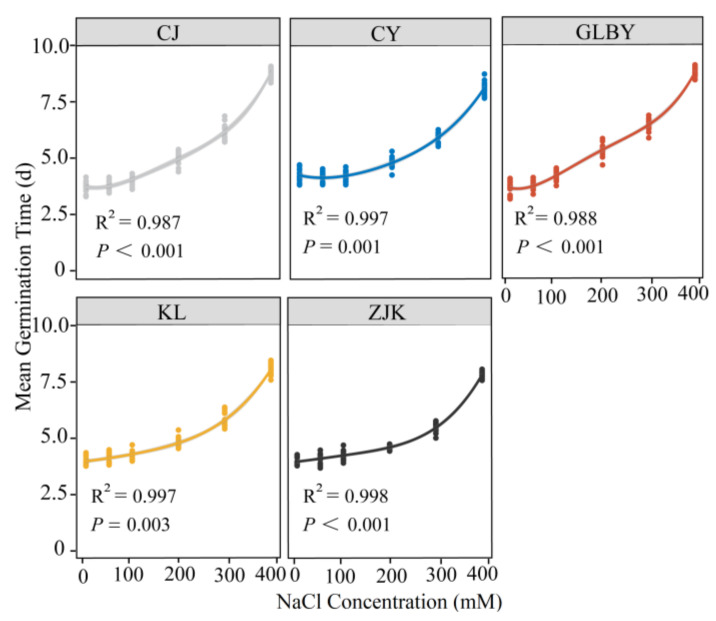
The effect of a NaCl gradient on the average germination time of *Solanum rostratum* Dunal (Solanaceae) seeds from different distribution areas. The lines represent the average time required for germination within 30 days after the start of the experiment. The points represent the standard errors of the means.

A linear model was fitted to the seed MGT as a function of the NaCl concentration. The results show that with the increase in NaCl concentration, the MGT of *S. rostratum* seeds from various areas showed a significant upward trend (*p* < 0.001). Under low NaCl stress (50 mM), except for CY, where the mean MGT was 4.08 d, the MGT of seeds from other distribution areas was less than 4 d. When the NaCl concentration reached a moderate level (200 mM), only seeds from GLBY had an MGT greater than five days (mean = 5.15 d), while seeds from other areas had MGT values less than five days. Under severe NaCl stress (300 mM), the MGT of GLBY (6.25 d) and CJ (6.07 d) exceeded six days, significantly higher than the MGT of seeds from other areas. Under the high concentration of NaCl (400 mM), the MGT of seeds from KL and ZJK was less than eight days, significantly less than that of seeds from other areas under the same salt gradient. Specifically, the response of seed MGT in seeds from the KL (R^2^ = 0.997, *p* = 0.003) and ZJK (R^2^ = 0.998, *p* < 0.001) areas to NaCl stress was relatively mild, while the seeds from the GLBY (R^2^ = 0.988, *p* < 0.001) and CJ (R^2^ = 0.987, *p* < 0.001) areas were more sensitive to NaCl stress (Figure 8).

### 3.4. The Effect of Osmotic Potential on the Germination of S. rostratum Seeds from Different Areas

**Figure 9:**
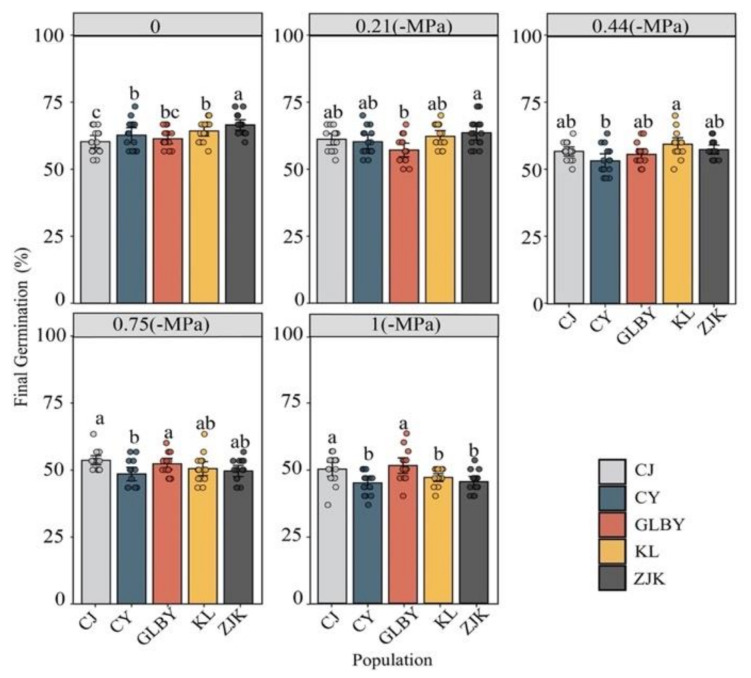
The effect of osmotic potential (water stress) on the final germination rates of *Solanum rostratum* Dunal (Solanaceae) seeds from different areas. Different letters represent significant differences between each distribution area under the same PEG stress conditions. The error bars at the top of the histograms represent the standard error. Dots indicate the original sample data.

The FGP of *S. rostratum* seeds from different areas significantly decreased with the increase in osmotic potential (*p* < 0.001) (Figure 9, Table 1). The results of the two-way ANOVA show that under stress-free (CK) conditions, the FGP (66.44%) of *S. rostratum* from ZJK was significantly higher than that in seeds from other areas. Under mild osmotic conditions (−0.21 MPa), the FGP of seeds from ZJK reached 63.56%, while the FGP of seeds from GLBY decreased to below 58%. Under moderate osmotic potential (−0.44 MPa), the FGP of seeds from KL was close to 60% (59.34%), significantly higher than that of seeds from CY. Under severe osmotic potential (−0.75 MPa), the FGP of *S. rostratum* seeds from the KL, CJ, and GLBY areas exceeded 50%, significantly higher than that in seeds from other areas. Under extreme osmotic potential simulated by PEG solution (−1 MPa), only the seeds from GLBY and CJ had FGP rates exceeding 50%, and these were significantly higher than those in seeds from other areas.

**Figure 10:**
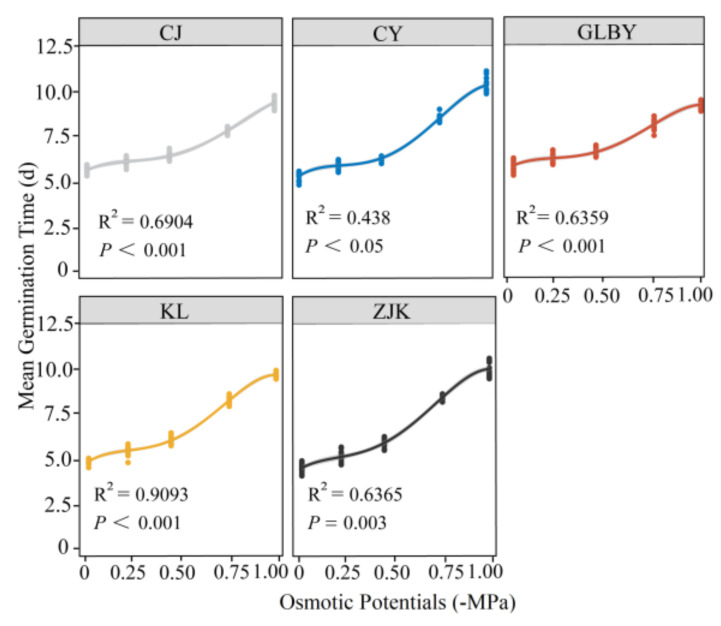
The effect of osmotic potential (water stress) on the average germination time of *Solanum rostratum* Dunal (Solanaceae) seeds from different areas. The lines represent the average time required for germination within 30 days after the start of the experiment. The points represent the standard errors of the means.

The mixed linear model fitting results for the response of seed MGT to changes in the PEG gradient showed that under no to mild (−0.21 MPa) osmotic stress conditions, the trend of MGT changes in the germination of seeds from the CJ area was not significant. Under severe osmotic stress (−0.75 MPa), the MGT of seeds from CJ was less than 8 d. The MGT of seeds from the CY area was sensitive to severe and extreme osmotic stress conditions. When the osmotic potential reached −1 MPa, the MGT of seeds from CY exceeded 10 days. The MGT of seeds from GLBY showed a relatively gentle increasing trend with the osmotic stress gradient. When the potential reached −1 MPa, the MGT of seeds from GLBY was less than nine days, significantly lower than the values for seeds from other distribution areas, indicating strong tolerance to severe osmotic stress. Under both CK and mild osmotic stress conditions, the MGT of seeds from KL was significantly lower than that of seeds from other areas. Under severe osmotic stress (−0.75 MPa) conditions, the MGT of seeds from KL was greater than eight days, showing a significant increasing trend. Under osmotic conditions of 0–0.44 MPa, the change trend in the MGT of seeds from ZJK was not significant. Under severe osmotic stress conditions, the MGT of seeds from ZJK was greater than eight days, showing a significant increased trend. Overall, the seed MGT in the CY (R^2^ = 0.9438, *p* < 0.05), KL (R^2^ = 0.9093, *p* < 0.001), and ZJK (R^2^ = 0.6365, *p* = 0.003) areas significantly increased with the increase in the osmotic stress gradient, and the slope of the MGT curve was significantly greater than those of the GLBY (R^2^ = 0.6359, *p* < 0.001) and CJ (R^2^ = 0.6904, *p* < 0.001) areas (Figure 10). The MGT of *S. rostratum* seeds from the Xinjiang samples was remarkably higher than the seeds from other samples.

## 4. Discussion

### 4.1. Adaptive Changes in Seed Size of S. rostratum to Habitat Variation

Seed size is the foundation for in-depth research on seed dispersal, population energy allocation strategies, and convergent adaptation of different groups [57]. Seed size not only is an important measure of the redistribution of sexual reproductive energy in a population but also has important ecological significance in population dynamics and spatial distributions [58]. This study found significant differences in weight and size among seeds of *S. rostratum* from different areas. The GLBY area had the lowest seed weight and the smallest seed size, while the KL area had the highest seed weight and the largest seed size. Generally, larger seeds (in terms of weight and size) contain more energy resources and are more likely to adapt to environments with strong competition and scarce resources, and such adaptation is beneficial for successful seedling colonization and community establishment [59,60,61]. Small seeds have stronger dispersal ability, a factor that is beneficial for expanding the distribution range of the population and occupying a dominant position in the community [62,63], thereby increasing the probability of successful invasion.

The seed size of *S. rostratum* varied among distribution areas, reflecting adaptation to heterogeneous environments. In the KL area, *S. rostratum* grows on a semi-arid seasonal riverbed with good water and thermal conditions, but not in the agricultural pastoral transitional zone where human activities have had a significant impact on the environment. According to field community surveys, the plant species richness in the KL area is relatively high, with interspecific competition between plant species. The seeds of *S. rostratum* in this area had greater weight and could easily be carried by livestock during grazing operations or spread through feces, increasing the seed bank in the soil. GLBY is in an extremely hot and arid area that has the lowest precipitation in China. The area has an average of 84 days with strong winds and an average annual wind speed of 8 m/s, making the area known as a “wind reservoir” or “fire island”. This area featured smaller and lower weight seeds that would be conducive to seed propagation under windy conditions. This result was consistent with previous research on the adaptability of invasive plant seed size to large-scale environmental differences [64,65].

In addition, research has suggested that larger seeds are beneficial for establishing larger seedlings after germination to compete for resources in the community [61]. When subjected to extreme heat stress, smaller seeds tend to have higher germination ability and stress resistance [66]. This can explain why in communities with high species richness, the mass of *S. rostratum* seeds was larger, while in high-temperature and arid environments, the mass of *S. rostratum* seeds was smaller. Large seeds can better ensure seed germination and seedling growth, while small seeds can ensure propagation and may be more suitable for invading the new environment [67]. Overall, the results for the mass and size of *S. rostratum* seeds in different areas indicate that *S. rostratum* has adapted to environmental heterogeneity and climate change.

### 4.2. The Response of S. rostratum Seed Germination Under Constant and Alternating Temperatures Gradients

For invasive plants, the germination ability of seeds under low temperatures can promote earlier germination, thus giving the species a dominant ecological niche advantage. The ability to germinate under high temperatures promotes the germination of invasive plants throughout the entire growing season, thereby increasing their success rate in “safe locations” [68]. Temperature factors significantly and positively affect seed germination [36], and temperature plays a dominant role in seed germination performance and geographical distribution patterns [34,69,70,71]. Our research shows that when under low-temperature conditions, the germination rate of *S. rostratum* seeds from the KL area exceeded 50%, and the average germination time of *S. rostratum* collected from the three distribution areas in Northeast China was less than seven days. The seed germination ability of *S. rostratum* in these areas under low-temperature conditions was stronger than that of seeds collected from the two distribution areas in Xinjiang. As the temperature increased, the germination rates of seeds from various regions significantly increased, and the average germination time also significantly shortened. At a constant temperature of 20 °C, the germination rate of seeds from ZJK was significantly higher than that of seeds from other distribution areas, and the MGT was the shortest. We believe that because the lowest annual average temperature among all sampling points is in the ZJK area, 20 °C is close to the average temperature of the local growing season (July). Therefore, the seeds from the ZJK distribution area were sensitive to temperature feedback and could germinate quickly. The maximum FGP and minimum MGT values of *S. rostratum* in each area were observed at 30 °C, with the FGP exceeding 90% and the mean MGT being only 3.36 days in seeds from the KL area. Note that at 35 °C, the FGP and MGT levels in seeds from the GLBY area located in an extremely arid region exceeded those under other temperature conditions. The seed FGP reached the highest level, and the MGT was the shortest. We believe that due to the GLBY area being in an extremely arid and high-temperature region, the seeds of *S. rostratum* growing in this area will experience high-temperature stress during the growing season (the extreme temperature at ground level in July was 49 °C). At the optimal germination temperature of 30 °C, seeds germinated rapidly, which was consistent with the theory proposed in previous studies that invasive alien species have a higher fitness advantage than resident communities; this is known as the “inherent superiority hypothesis” [72].

The germination rate of seeds from each area was the highest under 30/20 °C temperature conditions, with the FGP of seeds in two distribution areas in Xinjiang exceeding 80%, significantly higher than in seeds from other areas, while the MGT of the seeds was the lowest. The diurnal temperature treatment of 15/5 °C was close to the initial temperature in the three areas in Northeast China from March to April, and the diurnal temperature variation of 25/15 °C was close to the diurnal temperature difference during the peak period of plant development from May to June. Under these two temperature regimes, the seed FGP in the northeast (KL, CY, and ZJK) areas was significantly higher than in seeds from Xinjiang distribution areas, and the MGT was also significantly lower. Considering that the summer temperature in the GLBY area can reach 40 °C, a 38/28 °C variable temperature treatment was set to examine the adaptability of seed germination to high-temperature conditions in a heterogeneous habitat. Under these temperature conditions, the FGP of seeds from the Xinjiang areas (GLBY and CJ) was significantly higher than those of KL and ZJK seeds, and the MGT of seeds from these two areas was significantly lower than those of seeds from other areas. This indicates that the germination of *S. rostratum* seeds distributed in heterogeneous habitats is highly sensitive to temperature changes and that their potential germination niches are relatively broad. The seeds can quickly germinate in the early and late stages of seasonal environmental changes, occupying potential vacant niches and gaining a competitive advantage [24,73].

### 4.3. Response of S. rostratum Seed Germination to NaCl in Different Distribution Regions

Soil salinity is an important ecological factor affecting plant seed germination [74]. The three distribution areas in the northeast of this experiment are in the Horqin Sandy Land of the semi-arid farming pastoral ecotone in Northern China, an important livestock production area and commodity grain base, one of the areas with the most serious desertification and salinization [75]. The rapid formation of dominant species in the local plant community and the exclusion of local plants demonstrate the high ability to resist salt stress. NaCl was used to simulate salt stress to study the differences in the response of *S. rostratum* seeds from varied habitats. The results show that as the NaCl concentration increased, the germination rate of *S. rostratum* seeds significantly decreased. When the NaCl concentration was relatively mild (50–100 mM), the FGP of seeds from each area decreased, but the change in the MGT was not significant, indicating that mild salt stress affects the germination rate of *S. rostratum* seeds, while the effect on the germination response time was not as strong. With the increase in the salt concentration gradient, the FGP of seeds from the three areas in Northeast China was significantly higher than two areas in Xinjiang. The germination time required for seeds from Xinjiang was approximately five days. Under high salt concentrations, more than 50% of seeds from KL and ZJK germinated, and the germination time required was significantly shorter than that of seeds from Xinjiang, indicating that the germination of *S. rostratum* seeds from Xinjiang was more affected by salt stress than the northeast distributions. In extremely high salt-stress environments, the germination rate of *S. rostratum* seeds was significantly decreased, with only seeds from ZJK having a germination rate exceeding 40%. The FGP of seeds from CY was significantly lower than that of seeds from other areas under extreme salt stress conditions, but the difference in seed MGT between the CY distribution area and the other northeast areas was not significant (Figure 7), indicating that the seeds of *S. rostratum* in this region needed a longer time to germinate. We believe that this is because the CY sampling points were mostly in habitats such as livestock pens, farmland, and roadsides, areas that are strongly affected by human interference. The water and heat conditions within the habitat are unstable, and under extremely high salt stress conditions, the seeds in this area were more likely to enter a dormant state. This indicates that the germination of *S. rostratum* seeds has different response mechanisms to salt stress and that *S. rostratum* has strong salt stress tolerance. In the arid and semi-arid areas of Northern China, after the spring snowmelt and rainfall, the salt content on the soil surface will decrease and the osmotic potential will increase [76], conditions that may be more conducive to the rapid germination of *S. rostratum* seeds under saline–alkali soil conditions.

### 4.4. The Germination Response of S. rostratum Seeds from Different Areas to Osmotic Stress

Drought is one of the main characteristics of the Earth’s surface [77]. Drought stress is the most common form of plant stress, seriously affecting the processes of seed germination and seedling growth and hindering the normal metabolism of plants [78]. The response of seed germination to drought stress reflects a species’ adaptation mechanism to local environments. The study of the germination characteristics of invasive plants from heterogeneous habitats can explain their life history characteristics and provide a basis for clarifying the germination threshold of invasive plant seeds and future prevention and control [79,80]. Germination experiments were conducted on seeds of *S. rostratum* from different areas under varied osmotic potential, and the results show that the germination response of *S. rostratum* seeds to osmotic stress varied. Under mild osmotic stress (−0.21 MPa water potential), seeds from ZJK showed the highest values of FGP and the shortest MGT. When the osmotic stress reached a potential of −0.75 MPa, the seeds from the two areas in Xinjiang showed significant advantages in germination rate and germination time compared to the three distribution areas in Northeast China. With the increase in osmotic stress, more than 50% of seeds from Xinjiang germinated, and the MGT of seeds from GLBY did not exceed nine days, indicating extremely strong drought resistance. However, the FGP of seeds from CY was only 44%, and the MGT was longer than 10 days, indicating that osmotic stress has varied effects on the germination of *S. rostratum* seeds from different areas. This also indicates that *S. rostratum* seeds from heterogeneous habitats have different germination strategies under osmotic stress.

## 5. Conclusions

This study found significant differences in seed weight and size traits among five populations of *S. rostratum*, reflecting intraspecific variation in this species’ adaptation to heterogeneous habitats. While the germination of *S. rostratum* seeds demonstrated different response strategies to the various environmental stress factors, reflecting the ability of *S. rostratum* seeds to occupy germination ecological niches with low resource competition. Given the rapid changes in climate, understanding the germination behavior of alien invasive plants under natural conditions is crucial for predicting future plant community dynamics. Studying the differences in seed germination of *S. rostratum* is crucial for developing sustainable control strategies and restoration measures for invasive species. In response to the wide range of suitable biological flora and serious ecological hazards in arid and semi-arid regions of China, comprehensive and strict monitoring of potential invasive areas of *S. rostratum* should be implemented to ensure the ecological security of oasis agriculture and maintain local ecological diversity.

## Figures and Tables

**Table 1:** Two-way ANOVA for the final germination percentage of *Solanum rostratum* Dunal (Solanaceae) seeds from different populations. The effects of temperature, salt stress (NaCl stress), and osmotic potential (water stress) are shown.

Experiment	*df*	*F*-Value	*p*-Value
Constant temperature			
Plant	4	4.395	0.00172 **
Temperature	5	843.123	<0.0001 ***
Plant * temperature	20	1.797	0.00189 **
Variable temperature			
Plant	4	4.178	0.037 *
Gradient	3	916.463	<0.0001 ***
Plant * temperature	12	6.317	<0.0001 ***
NaCl stress			
Plant	4	7.369	<0.0001 ***
Gradient	5	1426.711	<0.0001 ***
Plant * gradient	20	4.852	<0.0001 ***
PEG stress			
Plant	4	4.752	<0.0001 ***
Gradient	4	154.389	<0.0001 ***
Plant * gradient	16	4.252	<0.0001 ***

Significance codes: 0, ***’; 0.001, **’; 0.01, *’.

## Data Availability

The original contributions presented in the study are included in the article. Further inquiries can be directed to the corresponding author.

## Abbreviations

ANOVA	One-way analysis of variance
Two-way ANOVA	Two-way analysis of variance
CMDSC	National Meteorological Science Data Center of China
FGP	Final germination percentage
MGT	Mean germination time
NIEER	Northwest Institute of Ecological Environment and Resources
PCA	Principal component analysis
PEG	Polyethylene glycol

## Appendix A

**Table A1:** Geographic locations and climate of five populations of *S. rostratum* used in this research.

Code	Population	Latitude (° N) Longitude (° E)	Altitude (m)	Habitats	MAT (°C, 2018)	AET (°C, 2018)	MAP (mm)	Seed Collection Time
CJ	Changji, Xinjiang	43.78–43.97° N 87.41–87.33° E	759–811	Farmland\roadside\irrigation canal	12.6 °C (April) 27.6 °C (July)	−28.7 °C (January) 43.2 °C (July)	127.9	August 28 September 7 October 5
GLBY	GuoLebuyi, Xinjiang	42.81–42.85° N 88.61–88.62° E	5–11	Farmland\irrigation canal\barren land	21.5 °C (April) 37.3 °C (July)	−10.4 °C (January) 49 °C (July)	7.7	August 25 September 10 October 3
KL	Kailu, Inner Mongolia	43.48–43.56° N 121.08–120.94° E	251–282	Grassland\river course in dry season	11.4 °C (April) 26.4 °C (July)	−13 °C (January) 27 °C (July)	397.6	August 23 September 6 October 3
ZJK	Zhangjiakou, Hebei	39.83–41.11° N 114.47–114.71° E	840–1411	Arboretum\farmland\greenbelt	9.8 °C (April) 21.4 °C (July)	−9.4 °C (January) 39.1 °C (July)	401.2	August 26 September 9 October 6
CY	Chaoyang, Liaoning	41.21–41.24° N 120.06–120.37° E	210–317	Livestock pens\farmland\roadside	13.3 °C (April) 26.5 °C (July)	−18.9 °C (January) 39.6 °C (May)	261.1	August 24 September 7 October 4

MAT: mean annual temperature in 2018; AET: annual extreme temperature in 2018; MAP: mean annual precipitation in 2018.
